# An online survey capturing the views of stakeholders on primary school food systems across the four UK nations

**DOI:** 10.1186/s12889-024-18149-x

**Published:** 2024-03-06

**Authors:** S. Spence, L. McSweeney, J. V. Woodside, D. Schliemann, on behalf of the GENIUS Network

**Affiliations:** 1 Human Nutrition and Exercise Research Centre, Population Health Sciences Institute, Faculty of Medical Sciences, Newcastle University, Newcastle upon Tyne, NE2 4HH UK; 2 Centre for Public Health, School of Medicine, Dentistry and Biomedical Sciences, Queen’s University Belfast, Belfast, BT12 6BJ UK

**Keywords:** Primary schools, School food systems, School food, Stakeholders

## Abstract

**Background:**

In 2020, the Generating Excellent Nutrition in UK Schools (GENIUS) Network was established to develop an understanding of the school food system across the four UK nations. This study explores stakeholders’ views (headteachers, teachers, parents and pupils) on what works well, the challenges, and what an ideal primary school food system includes.

**Methods:**

An online ‘School Food Survey’ was created in Qualtrics XM including closed and open-ended questions about the primary school food system. The Qualtrics link was distributed to stakeholders with an interest in school food through key contacts and networks across the four UK nations (21st June to 21st July and September 2021). Responses from the open-ended questions were exported from Qualtrics into Excel and analysed using SPSS. Aspects of qualitative content analysis were applied to summarise, code and quantify responses. Identified codes were entered by stakeholder, for example, parents and their response to the question into a Matrix table to allow identification of categories, themes and interpretation.

**Results:**

A total of 509 participants completed the survey: most participants were from Scotland (*n* = 281; 55%) and England (*n* = 213; 42%) and were parents (*n* = 394). There were some consistent views across stakeholder responses, for example, the range of healthy options, costs, and portion sizes offered to pupils. Parents views varied, with some expressing the range of healthy options worked well and others reporting too many unhealthy choices. The cost of school food and school food funding presented challenges for both parents and schools. For parents, an ideal school food system would include a wide variety of fresh healthy food choices that were made on site, use quality produce, be inclusive for all cultures and diets, and provide food portion sizes appropriate for pupils ages.

**Conclusions:**

The findings iterate the diversity and some inconsistencies between stakeholders, emphasising the complexity and competing tensions school food systems encounter. Parental involvement and consideration of school-level and national factors are important when identifying challenges, what works well and describing an ideal primary school food system.

## Background

Schools are often considered as ‘ideal settings’ to improve children and young people’s (CAYP) dietary intakes. Schools provide an environment where large numbers of CAYP attend daily potentially consuming food from breakfast to after-school time. This allows several time-points across the school day to influence CAYP’s dietary intakes. Over the years, numerous United Kingdom (UK) government reports have cited the school’s role in CAYP’s dietary intakes [1–4].

Furthermore, in the UK, there is legislation on the foods and drinks that can be served in primary schools across the whole school day [5], however, there are discrepancies across the four UK nations. England and Northern Ireland (NI) only have food- and drink-based standards, while Scotland and Wales have both food- and drink-based and nutrient-based standards, for example, maximum and minimum requirements for macro- and micro-nutrients [6]. In addition to legislation, there are non-legislated resources schools can use to improve what children and young people consume and learn about food in schools. For example, the School Food Plan is a UK web-based resource for schools that includes 17 actions (for example, food and nutrition training for headteachers, share what works well) [7], and globally, the World Health Organisation (WHO) provides web-based resources to enable schools to become ‘*health-promoting schools*’, including a focus on nutrition [8]. These resources are not legislation, therefore, no monitoring exists on the use by schools. More recently the CONNECTS-Food study has developed a free online resource to help primary schools develop their whole school approaches to school food. This resource includes a self-review tool for primary schools to identify where changes can be implemented, ideas on how to do this, and also, monitor any changes made [9].

In 2020, the Generating Excellent Nutrition in UK Schools (GENIUS) Network was established and funded by a UK Prevention Research Partnership (UKPRP) Network grant [10]. The network brought together researchers from a range of backgrounds and project partners actively involved in school food provision *(*i.e.*,* local government, catering providers) to collaborate on various activities with the key objective of developing an improved understanding of the school food system across the four UK nations [10]. As part of this, an online survey was created to explore stakeholders views (headteachers, teachers, parents and pupils) of the school food system. The school food system was defined as food and drink available across the whole school day, school food policies, food waste and other sustainability issues, procurement, school gardens, food provided at school events, food education and other activities that integrate with food culture and the environment. This definition was developed specifically for this project and by the GENIUS network. While some UK studies have considered stakeholder views on school food, these tend to be limited to a local area and by participant numbers.^(11; 12)^ Both of these studies explored the views of pupils and school staff, though were limited to the North/North-East of England. Day et al., (2015) [11] focused on primary schools, a key finding was pupils wanted increased food choice and variety. McSweeney et al., (2019) [12] focused on secondary school-aged pupils, a key finding was that whilst pupils are aware of healthy options they like to purchase hand-held, ‘grab and go’ items.

To date, we are unaware of any UK study that has sought to capture different stakeholder views (headteachers, teachers, parents, pupils) with representation from across the four UK nations. This analysis aimed to explore the views of stakeholders on the primary school food system across the four UK nations on what works well, the challenges faced and, what stakeholders consider to be an ideal school food system.

## Methods

### Ethics

Post-discussion with the Faculty of Medicine Health and Life Sciences Research Ethics Committee, Queen’s University Belfast, the included survey questions constituted Patient and Public Involvement (PPI) and did not require ethical approval. As the Ethics Committee deemed this did not require ethics - this was waivered by the named Institution (Queen’s University Belfast), therefore, we did not pursue written consent from stakeholders. Participants were clearly informed what the responses would be used for i.e., identifying areas for future research or to identify or share areas of good practice. Consent was assumed if an individual continued to complete the questionnaire after reading the statement on what their responses would be used for. No identifiable data was collected.

### Survey design

An online ‘School Food Survey’ was created in Qualtrics XM (Qualtrics, Provo, UT) [13] that included closed and open-ended questions about the school food system across the UK. This online survey was developed in collaboration with members of the School Food Review working group who also sought to gather information from school food stakeholders related to caterer responses to COVID-19 and the respective changes caterers implemented. The School Food Review working group is a body of individuals from various organisations (i.e., third sector, catering) that seek to improve school food. The survey incorporated a range of questions, along with, a skip logic function to enable respondents to move forward in the survey depending on a response given. As an example, respondents were asked about their role in the school food system (e.g., teacher, parent) and dependent on their response, this function forwarded them to specific questions relevant for them. The in-progress function allowed respondents to return and complete the survey at a later stage, though this was limited to a month. Filtering options were inbuilt into the Qualtrics survey to enable analysis of subsets of respondent’s data. The survey was tested with several members of the GENIUS Network involved in developing the survey to ensure there was a logical flow to questions, length of time to complete, and to identify any problems while completing the survey (i.e., ability to skip question).

Several closed questions were included to reduce respondent burden and enable some contextual information (i.e.*,* geographic location, primary or secondary school, and role within the school food system (i.e.*,* teacher, parent). No individual level demographics were collected. Open-ended questions were included to enable participants to have the opportunity to provide more information and express their views. The questions asked were adapted based on the particular stakeholder role identified. For example, headteachers and teachers were asked (i) to share examples of ‘good practice’ in their school and (ii) what were the major challenges experienced in their school food system. Parents and pupils were asked (i) what works well in their school food system? (ii) what does not work well? and (iii) what an ideal school food system would look like? Examples of the questions asked to parents were: (i) What works well within the current school food system at the school your child attends? (ii) What does NOT work well within the current school food system at the school your child attends? iii) What do you consider as priority areas for change within the school food system at the school your child attends? (iv) Of these, what is the greatest priority area at the school your child attends? (v) What would an ideal school food system look like from your perspective? Examples for pupils included: (i) What do you think works well (is good) in the school food system at your school? (ii) What do you think is NOT as good in the school food system at your school? (iii) Thinking about what you said worked well (was good) or is not as good in your school food system, what would you like to change? (iv) if you could only change one thing in your school food system, what would it be? (v) What would the best school food system look like to you?

### Recruitment and participants

The Qualtrics link was distributed to stakeholders with an interest in school food systems through key contacts and networks across the four UK nations (i.e., GENIUS network, social media, as well as via the networks of individual members of the School Food Review working group that had access to a wider range of schools etc. than the GENIUS network alone). The online survey was open from 21st June to 21st July 2021 and for the month of September (2021). Prospective participants received both the Qualtrics link and a short overview on (i) why the survey was being conducted (ii) what type of information/questions we were collecting/asking in the survey, (iii) what we meant by the term ‘school food system’, and (iv) how the responses would be used. Participants were free to decide if they wanted to complete the online survey or not, and if they wanted to complete all or part of the survey.

### Data analyses

Responses from the open-ended survey questions were exported from Qualtrics into an Excel spreadsheet and entered into SPSS to generate descriptive data. Stakeholder responses were organised according to question in a Microsoft Word document. A participant’s data was included in the analysis as long as they answered at least one question, they did not have to have completed all questions. Principles of qualitative content analysis were applied to code, summarise and quantify responses [14]. Summarised codes were entered by stakeholder/survey question into a matrix table to allow identification of categories (to deal with the large number of single item responses, categories were only generated from two or more stakeholder codes). One aspect of qualitative content analysis is to assign inductive categories to text to develop meaning/patterns from the text being analysed [15]. Secondly, to provide a more in-depth insight of the findings, and to help answer the survey questions, themes were identified from the responses, codes and categories using thematic analysis [16]. Team members met on a regular basis for quality control to discuss and agree on codes, categories and themes.

## Results

A total of 509 participants completed the survey across the four UK nations: most participants that responded were from Scotland (*n* = 281; 55%) and England (*n* = 213; 42%) (Table 1). Parents were the main stakeholders to complete the survey (*n* = 394), followed by headteachers (*n* = 60), teachers (*n* = 29) and pupils (*n* = 23). The results are presented in two sections, due to the differing numbers of responses, firstly, for headteachers, teachers and pupils and secondly, for parents. As most responses were from parents, a more detailed reporting of the results focused on parental responses.

**Table 1:** Number of participating stakeholders by UK Nation

Stakeholders	Nation			
	England	Scotland	Wales	Northern Ireland (NI)
(*n =* 509)	(*n =* 213; 42%)	(*n =* 281; 55%)	(n = 5; 1%)	(*n* = 10; 2%)
Headteachers	54	6	0	0
Teachers	22	7	3	0
Parents	121	261	2	10
Pupils	16	7	0	0

### Headteachers, teachers and pupils

Due to the small numbers of respondents from Wales and NI, the analysis does not differentiate by nation. Tables 2, 3 and 4 display the data categories and the tally (%) for each category by each survey question and stakeholder, followed by the identified themes. Selected illustrated quotes are provided from each of the three stakeholders.

**Table 2:** Categories and tally counts of headteacher responses

Survey question	Category	Tally	%
What are some examples of good practice in the school food system you are aware of? (*n* = 40 eligible responses)	Healthy food/choices	11	28
	Availability of fruit and veg	9	23
	Encouraging children to try new tastes/textures	7	18
	Healthy eating whole school ethos	7	18
	Breakfast clubs	6	15
	Flexible/ trained and communication/ consultation of providers/ kitchens	6	15
	Variety	4	10
What are the major challenges you or others face when it comes to the school food system? (*n* = 46 eligible responses)	Cost/funding to parents/school	21	47
	Unsatisfactory catering providers/staff	10	22
	Food quality	8	17
	Children not liking/trying foods	7	15
	Food access during holidays/Covid closures	3	7
	Unhealthy packed lunches from home	2	4
	Portion sizes	2	4
	None	2	4

**Table 3:** Categories and tally counts of teacher responses

Survey question	Category	Tally	%
What are some examples of good practice in the school food system you are aware of? (*n* = 19 eligible responses)	Good choice/variety of foods	8	42
	Provision of fruit (free for some)	6	32
	Breakfast club/provision	4	21
	Pre-ordering of meals from home	4	21
	None	2	11
What are the major challenges you or others face when it comes to the school food system? (*n* = 24 eligible responses)	Children’s reluctance to eat/try foods	8	33
	Portion sizes	7	29
	Packed lunches	4	17
	Cost/funding	4	17
	Time to eat	4	17
	Waste	3	13

**Table 4:** Categories and tally counts of pupil responses

Survey question	Category	Tally	%
What works well (*n* = 18 eligible responses)	Delicious/ good food/ healthy	7	39
	Cashless payment system	4	21
	None/ nothing	4	21
	Hot meals	2	11
	Choice	2	11
What does not work well? (*n* = 19 eligible responses)	Lack of variety/choice/bad food	18	95
	Over-priced/ expensive	7	37
	Long queues	2	11
	Lack of breakfast club	2	11
What would be an ideal food system? (*n* = 17 eligible responses)	More food variety/ better options	11	65
	Cheaper options/ better value	4	21

#### Headteachers

Responses were received from 60 headteachers from England and Scotland (Table 2).

Nine themes were identified from the headteacher responses:

- A range of healthy options are available to pupils including breakfast clubs.
- Schools encourage a whole school healthy eating ethos.
- Pupils are encouraged to try new and unfamiliar foods but can be reluctant.
- Good working relationships with catering providers and staff highlighted as important.
- Costs and funding of school foods presents challenges to both schools and parents.
- Unsatisfactory catering providers/staff can be an issue.
- Schools are concerned over pupil access to food during school holidays and the recent Covid-19 closures.
- Portion sizes are not always age appropriate.
- Ensuring that packed lunches brought from home are healthy can be challenging.

#### Teachers

Responses were received from 30 teachers from England, Scotland and Wales (Table 3).

Six themes were also identified from the teacher responses:

- Pupils are provided with good food choice, including breakfast, fruit snacks and lunch.
- Getting pupils to try/eat new foods can be challenging and can lead to waste.
- Portion sizes are not always age appropriate.
- Ensuring that packed lunches brought from home are healthy can be challenging.
- Costs and funding of school foods presents challenges to both schools and parents.
- Daily time constraints impact length of time pupils have in dining room.

#### Pupils

Responses were received from 23 pupils from England and Scotland (Table 4).

Six themes were identified from the pupil responses:

- Pupils were provided with a choice of delicious, healthy food with hot meal options.
- The use of cashless payment systems was liked.
- There could be a lack of variety or ‘bad’ food options available to pupils.
- Some options were felt to be expensive and not good value.
- Long queues impacted pupils’ dining experience.
- Breakfast clubs were not available to all pupils.

It is evident across stakeholder responses that they felt pupils were provided with a range of healthy choices throughout the school day. Pupils did, however, highlight there could also be a lack of variety or ‘bad’ foods:

“*There is not a lot of variety, for an example you can only have a few fillings in your panini and wraps” (pupil).*

Breakfast clubs not being available to all was described. Staff reported that portion sizes could be age-inappropriate:

*“Portion size means many older children do not select meals [set meal of the day], they are still hungry after” (teacher)*.

Both headteachers and teachers described how encouraging pupils to try new or unfamiliar foods could be challenging. The types of foods pupils brought to school in packed lunches did not always meet the school’s healthy eating guidelines:

*“Some parents prefer to give packed lunches so children can eat less healthy choices” (headteacher)*.

Teachers and headteachers acknowledged that the costs/funding of school foods was of concern and impacted both schools and parents. Pupils too, felt that some options were not good value for money. Ensuring a good working relationship with catering providers/staff was a priority for headteachers, but some mentioned issues with unsatisfactory providers:

“*Local Authority food providers seem to focus on providing a cheap meal rather than healthy, nutritionally balanced meal” (headteacher)*.

Pupils also mentioned the negative impact of queuing on their dining experience but liked a cashless payment system.

### Parents

Categories and tallies generated from the parent responses are presented in Table 5. As parent responses were highest in Scotland (*n* = 261) and England (*n* = 121), but low in Wales (*n* = 2) and Northern Ireland (*n* = 10), responses have been analysed and presented for the four UK nations combined.

**Table 5:** Categories and tally counts of parent responses

Survey question	Category	Tally	%
What works well? (*n* = 363 eligible responses)	Selection/ variety of choices	105	29
	Ease of ordering/ payment	72	20
	Ability to see menu/ order in advance	61	17
	Free school meals	37	10
	Child-friendly/ liked foods	20	6
	Hot and cold choices	23	6
	Halal and vegetarian options	11	3
	Not much/ nothing	10	3
	Availability of packed lunch option	10	3
	School healthy eating policy/ education	7	2
	Healthy/ balanced meals/ food	12	3
	School lunch	9	2
	Breakfast club	6	2
	Everything/ all works well	6	2
	Quality of food	3	1
What does not work well? (*n* = 378 eligible responses)	Poor choices/ variety/ options	84	22
	Poor food quality	48	13
	Small portion sizes	42	11
	Children not liking choices/complaining	42	11
	Lack of special dietary options	25	7
	Too many unhealthy choices/ junk foods	31	8
	Food running out/ pre-ordered choice not available	22	6
	Lack of healthy choice	18	5
	Time to eat	18	5
	Lack of allergen/ nutrition information	12	3
	Unavailability of salad/veg sides	16	4
	Lack of school healthy eating policy	13	4
	Cost	15	4
	Pre-ordering system/ App	14	4
	Inflexible menu ordering system	15	4
	Availability of snacks/ fruit	10	3
	Nothing/ not sure	10	3
	Plastic waste	8	2
	Changes made during Covid restrictions	6	2
	Lack of information about/ on menus	7	2
	Repetitive menus	4	1
	Lunch hall environment	4	1
	No juice/ alternatives to milk/ water	3	1
	Food being reheated	5	1
	Too many vegetarian options	4	1
	Long queues	5	1
	No breakfast club provision/ unaffordable	3	1
	Ham sandwiches no longer allowed	5	1
What would be an ideal school food system? (*n* = 304 eligible responses)	Range of healthy/ balanced options	73	24
	Variety	37	12
	Fresh/ home-cooked food/ made on site	37	12
	Provision of fruit and veg	27	9
	Quality produce/ ingredients	25	8
	Pre-ordering App/ cashless system	24	8
	Less convenience/ processed foods	22	7
	Current system fine/ no changes	19	6
	Child-centred approach/ consultation of children	16	5
	Not reheated/ gone cold food	16	5
	Caters for all dietary requirements	12	4
	Good/ age-appropriate portion sizes	13	4
	Whole school approach to health eating	12	4
	Free school meals for all	13	4
	Provision of sandwich options	6	3
	Don’t know	6	3
	Calm, social dining rooms/ eating ethos	6	3
	Food that children like/will eat	9	3
	Provision of healthy snacks	8	3
	Sustainable/ ethical ingredients/ food	6	3
	Choice of sides/ desserts	8	3
	Soup option	5	2
	Breakfast clubs/ provision	7	2
	Buffet style of foods	5	2
	Adequate time to eat	6	2
	Options to mix/match menu choices	4	1
	Growing fruit and veg in school gardens	4	1
	Reasonable/ manageable costs	4	1
	Trained teachers/ staff in healthy eating/ cultural diversity	4	1
	Food that is edible	4	1

In response to the question ‘what works well?’ the highest number of responses related to ‘selection/variety of choices’, the least responses related to ‘healthy eating policy/education’ and ‘quality of food’. Paradoxically, the opposing view, ‘Poor choices/variety/options’, was also the highest response for ‘what does not work well?’, with the least responses pertaining to categories, such as, ‘too many vegetarian options’, ‘no breakfast club provision/unaffordable’ and ‘no juice/alternatives to milk/water’. The question, ‘what would be an ideal school food system?’ elicited similar responses relating to food choice, however, the emphasis for an ideal system was more on providing ‘healthy/balanced options’ and ‘fresh/home cooked/made on site meals’ as opposed to just providing variety/options as reported in the previous two questions. The least frequent responses pertained to: ‘growing fruit and vegetables in school gardens’, ‘reasonable/ manageable costs’, ‘trained teachers/staff in healthy eating/cultural diversity’, and ‘food that is edible’.

Table 6 outlines the themes identified from the parent findings from each of the three questions.

**Table 6:** Themes identified from parent responses combined across the UK nations

What works well?	What does not work well?	What would an ideal food system look like?
● Food offered is healthy/balanced with variety of hot/cold child-friendly options ● Good options for those with additional dietary requirements ● Parents like access to menus and pre-order school lunches ● Straightforward, online pre-payment systems ● Some awareness of their child’s school healthy eating policy or that pupils receive healthy eating education as part of curriculum ● Free school meals for the younger children	● Pupils given too many unhealthy ‘junk’ food choices ● Lack of healthy options available ● Lunch choices can be restrictive and lacking in special dietary options/alternative options ● Pupils complain of not liking/enjoying food provided and report small portions ● Inflexible pre-ordering system ● Sometimes pre-ordered choice is not available on the day. ● Menus do not provide enough information including allergen and nutritional information and often repetitive ● Pupils experience long queues and not enough time to eat ● Lack of school healthy eating policy ● Too much reliance on single-use plastic ● Cost of school lunches and breakfast clubs	● School food would focus on a wide range of healthy, fresh, home-cooked options prepared in school kitchens. ● School caterers would use quality, sustainable/ethical ingredients with schools growing own fruit and vegetables ● School lunch pre-ordering system would be a cashless payment App ● Parent/child would have the option to mix and match food items instead of being restricted to fixed menus ● A whole school approach to healthy eating, with a focus on pupil involvement and consultation ● School lunches free for all or provided for a manageable cost to families ● School lunches would cater for all dietary requirements whilst serving age-appropriate portions. ● School staff would be trained in healthy eating/cultural diversity ● The dining hall would have a calm, social atmosphere with pupils having adequate time to eat without feeling rushed

Due to the high number of themes, the results are presented by question type; illustrative quotes from parents are provided for each question:

*What works well?’*

Parents report that a wide choice and variety of healthy options is available for the pupils including those from diverse backgrounds:

‘*Different variety of food as non-veg, veg and halal.’* *‘Good variation in hot meals each day. Like that there is always soup available too - healthy and popular!’*

*What does not work well?*

However, conversely when asked ‘what does not work well’, some parents reported a lack of variety, choice, too many processed and junk food items, and the issue of compliance with school food standards:

*‘Food is not of a high quality, does not provide well for alternative diets. Portion size [sic] not enough, seems to be no effort to reduce sugar or use more healthy ways to add sweetness to puddings or snacks.’*

‘*The school meals are not School Food Standard compliant. I’ve raised it a few times and they change for a while and then the following term go back to processed potato products 4x a week. There are only ever 3 vegetables used throughout the 3x week menu - and one of those is baked beans, which is even served with roast dinners. Sweet puddings served most days. I’m aware that these ‘may’ be fruit based and SFS compliant, but it’s really unhelpful in creating a level playing field when the packed lunch policy asks parents not to send cakes in’.*

Parents who had access to pre-order cashless payment systems appeared to appreciate the ease in which they could pre-order their child’s lunches. However, some applications were inflexible in what the parent/child wanted to order:

*‘Menus aren’t great, separate items would be better to select.’*

*‘It is difficult for them to mix and match food available, menu often set [sic].’*

It was also felt that menus could be lacking in nutrition and detailed dietary information:

*‘No nutrient information provided to parents to enable a fully balanced diet.’*

*‘Lack of information about ingredients, allergens, accompaniments and alternatives for allergies and intolerances.’*

Furthermore, parents reported that pre-ordered food was not always available or had run out before the child had got their lunch:

*‘Due to staggered lunches my older child regularly doesn’t get the food I pre-ordered and paid for as there is not any left. I find this hard to understand. Surely the school should make enough for the orders that day. This happens too often.’*

There was some concern that portion sizes were not appropriately measured with younger and older children receiving similar amounts:

*‘My child states that the portion for lunch is small and she is still hungry.’*

*‘Portion sizes are not enough for older children e.g. year 5 and 6 as these are the same portion sizes as for reception children.’*

*What would an ideal food system look like?*

Parents were keen that in an ‘ideal school food system’, school lunches would be home-cooked on school premises using quality, fresh, healthy ingredients:

‘*Grown locally, cooked on the premises. Homemade goujons. Homemade steak pie. Soups and healthy puddings. Fresh fruit and crudités.’*

*‘Homemade, non-processed, balanced meals on offer.’*

For those parents in England and Scotland where universal free school meals are provided for some children, this was appreciated. Some parents felt this offer should be extended to all primary school aged children:

‘*Extend free school meals to ALL children.’*

*‘Free school meals for all Primary children.’*

Parents demonstrated a desire for their child’s school to have a holistic approach to healthy eating with policies in place to cover all times of the day and activities:

*‘Teachers have proper food knowledge and lead by example by eating with the children, reference good food and making good food the natural choice. The food provided in school is nutritious and delicious. Children have access to healthy food before, during and after school as required. There are school gardens and orchards and learning food growing and cooking skills is integrated into the curriculum.’*

*‘They [pupils] need to be active citizens in their school food system. Schools should be the gold standard of what we want to see across the rest of society and we need to develop a culture in a generation of children where enjoying eating healthily, enjoying eating fruits, vegetables and new foods is the norm.’*

Also highlighted, was the request for a calmer, less frantic dining hall experience where pupils had time to eat their meal in a sociable environment:

*‘The children don’t always have enough time to finish their dinner however, which isn’t ideal.’*

*‘A noisy, messy, and rushed dining environment.’*

Moreover, ensuring schools are places of inclusivity to all for health and wellbeing:

*‘One [ideal school food system] that ensures that every child has the opportunity to access good quality healthy food choices.’*

*‘Caters for every religion background, [and] dietary needs.’*

## Discussion

### Summary of key findings

The aim of the survey was to collect stakeholder views on the primary school food system. However, as parents were the main responders, the more detailed analyses has focused on parents. The parent responses highlight they have lots of opinions and suggestions about what they feel works, does not work and what an ‘ideal school food system’ should look like.

There are some consistent views across stakeholders (headteachers, teachers and parents). These include, the range of healthy options, issues related to costs, and portion sizes. For parents, the views varied, some expressed the range of healthy options worked well, whereas others reported there were too many unhealthy choices. Similarly, for some parents the pre-ordering and cashless payment systems worked well, while for others, this required improvement. Cost had negative implications, headteachers and teachers noted costs and funding presented challenges for both schools and parents. Parents and pupils mentioned costs in relation to school food provision. Stakeholder responses on school healthy eating policies were inconsistent; headteachers responded that they encourage a healthy eating ethos, in contrast, some parents found healthy eating policies did not work well. The inconsistency in findings across stakeholder groups may be explained by school-level variation which we cannot capture. Some schools have additional school food policies other than the school-food standards required by legislation. This depends on the school leadership. Currently, there is no mandatory evaluation of school food compliance, therefore, the school food offer and quality may vary by school. In addition, there is variation in school food payment options (cashless systems verse payment). These issues may provide insight into the parent-level variation. Parents expressed an ideal school food system would include: a variety of fresh healthy food choices that were made on site, used quality produce, were inclusive for all cultures and diets, age-appropriate portions, and involved pupils. Parents focused less on wider issues such as the dining environment, school gardens, management costs, and staff training.

### Relationship to other studies

One large survey in the UK on parent views on primary and secondary school meals was undertaken by ParentPay, Cypad and Local Authorities Caters Association (LACA) 2021 [17]. Similar to our survey, most of their respondents were from England (87%). While the methods, questions and rationale were different, for example, the LACA study was limited to parent views on the school meal service and food on offer, there were some similar findings. These included: pre-ordering was important to parents, more than one in four felt a school meal was too expensive, and in relation to quality of the school meal ‘food variety’, ‘nutritional value’, and ‘food standards’ were important to parents. Also important to parents was the ability to see the menu and nutritional content, along with a picture of the food.

The issues related to food choice are not new, a study by Booth et al., (1990) [18] undertaken in two secondary schools in Nottinghamshire that considered the views of pupils on school food noted ‘variety’, ‘cost’ and ‘quantity’ as the most important factors. Although only a small number of pupils completed our survey, similar issues were reported. Other smaller studies exploring the views of pupils and parents on developing free school meals in Finland, found ‘variety of foods’, ‘wider selection of salads’ and ‘inclusion of favourite dishes’ as important aspects [19]. Furthermore, Day et al., (2015) [11] explored the views of pupils and catering staff in primary schools on aspects such as ‘healthfulness’, ‘food quality’, ‘food choice’ and ‘satisfaction’ with school meals, similarly, pupils reported they wanted increased ‘food choice’ and ‘variety’.

### Strengths and limitations

The use of an online survey had several advantages: lower costs, convenience for participants to complete at a suitable time, the design allowed participants to skip questions should they want to, there was no potential of interviewer/respondent bias and it provided an opportunity for stakeholders across the four UK nations to participate. There were several key limitations: lack of detailed responses were obtained for example, if a parent mentioned the school’s healthy eating policy did not work well there was no opportunity to probe responses for further understanding and clarification. There was also an inability to differentiate effects such as socio-economic, and stakeholder responses at a school level in the analysis. Whilst we used key contacts to distribute the survey link across the four UK nations, there were limited responses from stakeholders in Wales and NI. This lack of response by stakeholders in some UK nations meant we analysed the data combined across the four UK nations, and does not enable comparisons at national level. There was no mechanism to identify if a participant completed more than once. Pupils were primary school aged (aged 4-11y) and only a small number of pupils completed the survey, therefore, pupil views were under-represented. Stakeholders provided information freely in this survey and there is a risk of participation bias from more engaged stakeholders, or those that have strong views on the topic of school food. However, the data collected from parents provides some novelty in obtaining parental views.

## Conclusion, relevance to policy and practice and future research

The findings iterate both the diversity and some inconsistencies between stakeholder views, emphasising the complexity and competing tensions school food systems encounter. School food systems are further subject to different influences at the individual school level, highlighting the potential diversity in stakeholder views. For example, some of the diversity in parent views may be explained by the fact that some schools are doing better in relation to the school food system (i.e., pre-ordering systems and variety of healthy food choices) whilst other schools face challenges, despite school food standards. Across the four UK nations increased parental involvement and consideration of school-level and national factors are important when identifying challenges, what works well and describing an ideal primary school food system. Future research would benefit from understanding the variations between schools and factors that enable some schools to positively consider their school food system.

## Data Availability

The datasets generated and/or analysed during the current study are not publicly available as meaningful data are included but are available from the corresponding author on reasonable request.

## Abbreviations

CAYP: Children and young people
UK: United Kingdom
PPI: Patient and public involvement
UKPRP: UK Prevention Research Partnership
*n*: Number
NI: Northern Ireland
N/A: Not applicable
LACA: Local Authorities Caters Association
